# Non-continuum solvation using the EC-RISM method applied to predict tautomer ratios, p*K*_a_ and enantiomeric excess of alkylation reactions

**DOI:** 10.1186/1758-2946-4-S1-O9

**Published:** 2012-05-01

**Authors:** Jochen Heil, Roland Frach, Stefan M Kast

**Affiliations:** 1 Technische Universität Dortmund, Dortmund, 44221, Germany

## 

The three-dimensional “reference interaction site model” (3D-RISM) integral equation theory is a statistical-mechanical approach to predict liquid state structural and thermodynamic features. It is based on approximate solute-solvent correlation functions to be computed on a 3D grid as a function of the interaction potential between the solute and the solvent sites, circumventing the need of costly sampling of explicit solvent degrees of freedom. In combination with quantum-chemical calculations within the embedded cluster (EC-)RISM framework [1] the theory allows for studying chemical reactions in solution with an accuracy not reached by traditional continuum solvation methods. In particular, it improves upon dielectric continuum solvation by taking solvent granularity into account and also provides a means towards physically cavity formation and dispersion free Energies without introducing artificial boundaries and empirically fitted radii.

We outline the general framework and show application examples from p*K*_a_ and tautomeric ratio estimation [2] as well as enantiomeric excess prediction for stereoselective alkylation reactions in organic solvent.

## References

[B1] Kloss T Heil J Kast SM Quantum Chemistry in Solution by Combining 3D Integral Equation Theory with a Cluster Embedding Approach J Phys Chem B 2008 112 4337 4343 10.1021/jp710680m 18341326

[B2] Kast SM Heil J Güssregen S Schmidt KF Prediction of tautomer ratios by embedded-cluster integral equation theory J Comput-Aided Mol Des 2010 24 343 353 10.1007/s10822-010-9340-x 20352296

